# Pushing the endogenous envelope

**DOI:** 10.1098/rstb.2012.0506

**Published:** 2013-09-19

**Authors:** Jamie E. Henzy, Welkin E. Johnson

**Affiliations:** Biology Department, Boston College, Chestnut Hill, MA 02467, USA

**Keywords:** endogenous retrovirus, retrovirus, envelope, phylogenetics, transmembrane subunit

## Abstract

The majority of retroviral envelope glycoproteins characterized to date are typical of type I viral fusion proteins, having a receptor binding subunit associated with a fusion subunit. The fusion subunits of lentiviruses and alpha-, beta-, delta- and gammaretroviruses have a very conserved domain organization and conserved features of secondary structure, making them suitable for phylogenetic analyses. Such analyses, along with sequence comparisons, reveal evidence of numerous recombination events in which retroviruses have acquired envelope glycoproteins from heterologous sequences. Thus, the envelope gene (*env*) can have a history separate from that of the polymerase gene (*pol*), which is the most commonly used gene in phylogenetic analyses of retroviruses. Focusing on the fusion subunits of the genera listed above, we describe three distinct types of retroviral envelope glycoproteins, which we refer to as gamma-type, avian gamma-type and beta-type. By tracing these types within the ‘fossil record’ provided by endogenous retroviruses, we show that they have surprisingly distinct evolutionary histories and dynamics, with important implications for cross-species transmissions and the generation of novel lineages. These findings validate the utility of *env* sequences in contributing phylogenetic signal that enlarges our understanding of retrovirus evolution.

## 1. Introduction

Much of the reconstruction of retroviral lineages has centred on the well-conserved reverse transcriptase (RT) motif of the polymerase gene (*pol*) [1–5]. Its high level of sequence conservation facilitates the design of primers that cast a wide net, amplifying RT sequences from a wide range of vertebrates [6–9]. Additionally, conserved motifs allow easy alignment of even distantly related proteins, demonstrating, for example, the common ancestry of retroviruses and the many RT-containing elements found among various kingdoms of life [10]. Early RT-based analyses revealed the surprising fact that proviruses representing retroviruses estimated to have infected their hosts tens of millions of years ago or more harbour recognizable features of extant retroviruses, despite their notoriously high substitution rate [11–13]. While this situation leads evolutionists to ponder the ‘molecular clock’ conundrum [14], the fidelity of features over deep evolutionary time also gives us a set of data that can be analysed using phylogenetic methods, revealing evolutionary dynamics of retrovirus–host interactions.

Phylogenetic analysis of ERV RT sequences also reveals that ERVs cluster closely with genera of extant retroviruses. Phylogenetic trees combining RT sequences from both endogenous and exogenous retroviruses suggest that all known retroviruses can be grouped into three broad classes [4,15]: class I consists of gammaretroviruses, epsilonretroviruses and the ERVs that cluster along with them. Class II comprises the beta- and alpharetroviruses, and the lentiviruses, along with related ERVs. While no endogenous deltaretrovirus-like element has yet been discovered, an argument to include deltaretroviruses under the class II umbrella can be made based upon the catalytic site (YMDD) in RT, which matches that of the other class II members. Class III ERVs cluster with spumaretroviruses [16]—an ancient group of retroviruses that have largely co-speciated with their hosts [17].

Although phylogenetic work based on RT has its advantages, a disadvantage of using this most highly conserved region of the retroviral genome is that many finer distinctions between lineages are blurred. Additionally, just as organismal chromosomes can have complex independent histories, recombination uncouples the evolutionary history of different parts of the retroviral genome [18]; consequently, RT sequences reflect only one portion of the historical lineage giving rise to any given retrovirus genome. In this review, we explore some interesting details that analysis of endogenous retroviral *env* genes can provide, beyond those provided by the *pol* gene. When we allow *env* to tell its own story, new insights into retroviral evolution emerge, involving evidence and likelihood of cross-species transmissions, the evolution of new lineages and possibly genera, and even recombination between virus families that could generate new types of viruses.

## 2. Transmembrane subunit: features and function

For our purposes, we will focus on the ERVs of class I and II and their exogenous cousins, which encompass the *Orthoretrovirinae*, and include all currently recognized retroviral genera except the spumaretroviruses [19]. The fusion subunit of the envelope glycoproteins (Env) of most orthoretroviruses are class I viral fusion proteins (not to be confused with class I retroviruses) sharing structural and mechanistic features with filo-, paramyxo-, orthomyxo- and coronaviruses [20]. (Epsilonviruses are the exception—Env proteins representing this genus have not been characterized, but sequence analysis predicts a distinct structure. For these reasons, they will be omitted from this review.) Retroviral Env is synthesized as a polyprotein precursor that trimerizes in the endoplasmic reticulum and is cleaved by a host protease in the late Golgi apparatus. The result is a trimer of heterodimers, each consisting of two subunits—SU, for surface domain, and TM, for transmembrane domain—that either remain associated non-covalently, as in the case of betaretroviruses and lentiviruses, or by virtue of a single intersubunit disulfide bond, as found in gamma-, delta- and alpharetroviruses, and the subset of betaretroviruses formerly known as type D [21–26]. The Env trimers, anchored into the cellular membrane via the TM subunit, then traffic to the plasma membrane and stud the surface of the newly budding virus particles [27].

SU has maximum exposure to the host immune system, and includes the receptor binding domain (RBD); thus, it is under heavy adaptive pressure [28] and is poorly suited for phylogenetic analyses. TM, by contrast, is mostly shielded from the immune system by SU, and carries out the highly conserved, essential function of fusing the viral and host cell membranes during viral entry. Given the importance of this function, it is not surprising that the TM encoding portion of *env* is sufficiently conserved to be useful in phylogenetic analyses [16].

**Figure 1.**
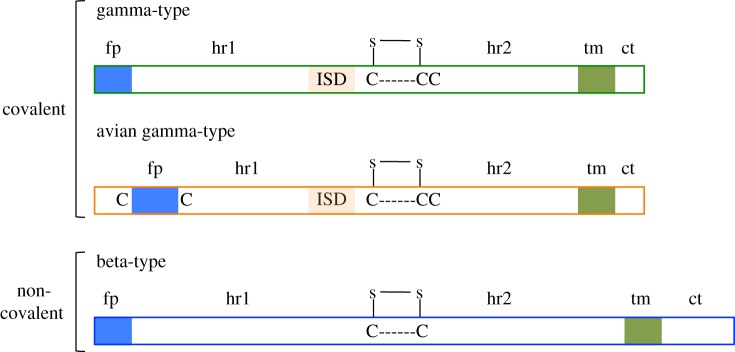
Three TM types found among the Orthoretroviridae. fp, fusion peptide; hr1 and hr2, heptad repeats 1 and 2; ISD, immunosuppressive domain; tm, transmembrane region; ct, cytoplasmic tail. The disulfide bonded loop is depicted above the cysteine motifs.

The essential functions of TM are reflected in a highly conserved domain organization (figure 1). The cleavage site between SU and TM consists of a polybasic motif (K/R–X–K/R–R) [29] and marks the beginning of the TM portion of the sequence. The TM sequence has two hydrophobic stretches that flank an ectodomain (the portion of TM exposed on the outside of the virion). The first hydrophobic stretch, at or near the N-terminus, constitutes the fusion peptide (fp), and the second is the transmembrane region (tm), by which TM is anchored into the viral membrane. Prominent features of the ectodomain include two heptad repeat regions (hr1 and hr2) flanking a stretch of residues that contains either two or three cysteines. The heptad repeats play a critical role in the dynamic rearrangement of the trimer during the process of fusion, and formation of the highly conserved coiled-coil structure that is found among many viral fusion proteins [30]. The ectodomain sequence of some retroviruses also includes a region known as the immunosuppressive domain (ISD)—a stretch of 20 amino acids immediately upstream of the first cysteine residue, recognizable by its conserved residues [16]. Finally, C-terminal to the transmembrane region is the cytoplasmic tail (CT), which is located on the cytoplasmic side of the cellular membrane and after assembly, on the interior side of the viral membrane. The CT can be highly variable both in length and sequence, even among retroviruses of the same genus.

Within the Env trimer at the surface of the virion, SU holds TM in a metastable conformation, by analogy with the ‘spring-loaded’ model ascribed to influenza haemagglutinin [31]. Upon binding to the receptor, a conformational change in SU exposes the fusion peptide of TM, which then inserts into the plasma membrane, either at the cell surface or within an endocytic compartment. TM subunits then fold into a highly stable structure consisting of a trimer of ‘hairpins’, in which the alpha-helical coiled-coils of hr1 and hr2 pack against one another. The energy released as TM trimers move from the metastable to the stable state drives the fusion of the cellular and viral membranes [32].

In addition to mediating fusion, TM also contributes to infection by other means. The ISD has been shown to inhibit lymphocyte proliferation [33] and allow escape from immune effectors of the innate and adaptive arms of the host immune system in a mouse model [34–37]. Some functions that have been associated with the CT include modulation of fusogenicity [38], interaction with cell signalling pathways [39,40], and possibly incorporation of Env in virus particles [41]. The membrane proximal external region (MPER) is a stretch of 30 residues immediately upstream of the transmembrane region that, in HIV-1, is thought to be important for Env incorporation into virions, as well as membrane disruption during fusion [42–44]. The cysteine pair in the ectodomain is highly conserved across the orthoretroviral TM (except in epsilonviruses). The cysteines are covalently bonded via a disulfide link, forming a loop within the TM ectodomain that is involved in interaction with SU. Studies have shown that elimination of the loop abrogates fusion [45].

## 3. Transmembrane types

Among the orthoretroviruses and related ERVS are found three types of envelope glycoproteins (again, the *env* of epsilonviruses is excluded). Each type is distinguished by features of secondary structure found in the TM amino acid sequence. The three TM types that are found among class I and II retroviruses are here referred to as the gamma-type, the avian gamma-type and the beta-type (figure 1). Importantly, the TM type of a given retrovirus does not always reflect its genus. For example, those members of the betaretrovirus genus formerly known as type D (i.e. Mason–Pfizer monkey virus, MPMV) possess gamma-type *env*, reflecting a recombinant origin [46].

### (a) Gamma-type and avian gamma-type

The gamma-type and avian gamma-type are found among retroviruses in which SU and TM are covalently linked. The gamma-type is representative of gammaretroviruses, deltaretroviruses and the recombinant betaretroviruses (those formerly known as type-D retroviruses); the avian gamma-type is a variant of the gamma-type that is, so far, found only among alpharetroviruses. Because the SU and TM subunits of these retroviruses are linked by a disulfide bond, a third cysteine is required (in addition to the pair that form the highly conserved intramolecular loop in the TM ectodomain) to participate in a disulfide bond formed with SU [21,25,47–49]. In both the gamma-type and the avian gamma-type TM, the three cysteines are found in a rigidly conserved CX_6_CC motif, immediately downstream of the ISD sequence [16]. The avian gamma-type differs from the gamma-type in that an internal fusion peptide is located approximately nine amino acids downstream of the cleavage site rather than at the N-terminus. Additionally, in the avian form, the fusion peptide is flanked by a pair of cysteines that form a disulfide bond with one another [50].

### (b) Beta-type

This type is found in retroviruses in which SU and TM are non-covalently associated—the non-recombinant betaretroviruses (those formerly known as type B) and lentiviruses. Because there is no intersubunit disulfide bond, only the two loop-forming cysteines are required in the ectodomain. The beta-type motif is CX*_n_*C, with the number of residues separating the cysteines varying from four to seven [26]. Additionally, the beta-type TM lacks a recognizable ISD sequence, and the MPER is longer by 20–30 amino acids than the corresponding region of the gamma-type [16].

For purposes of gaining inferences into retroviral evolution, we can ask: how are these envelope types related to each other? How is the beta-type, with its two-cysteine motif, related to the gamma-type, with its three-cysteine motif? Did one arise from the other, losing or gaining a cysteine, for example? The shared domain organization and common features allow us to construct reasonable alignments of gamma- and beta-types. However, it is important to note that, at the sequence level, these two types are so highly diverged that basic local alignment search tool (BLAST) [51] searches with the gamma-type TM sequence do not return any beta-type TM sequences and vice versa, despite the use of various parameters and datasets. Such divergence applies even when the ISD region is removed from the analysis [16], or when cysteines in the ectodomain have been mutated [26]. In fact, the genetic distance between the gamma-type and beta-type TM is great enough that it allows for the possibility that they were acquired from independent sources, and that their shared features are due to convergent evolution rather due to than common ancestry. This situation has implications for phylogenetic analyses, as alignments of non-homologous sequences will not reveal valid evolutionary relationships.

For retroviral fossil hunters, this high degree of divergence is fortuitous, because it allows even very degraded *env* sequences to be readily categorized, simply by examining whether a BLAST search using the sequence as a query turns up one or the other *env* type. Class I and class II RT sequences, by contrast, are much less divergent and are clearly homologous (e.g. using a class I ERV RT sequence as a BLAST query will readily return class II sequences).

Moreover, the fidelity of these TM types can be seen throughout the known ERV fossil record. RELIK—the oldest known endogenous lentivirus, thought to have infected the rabbit lineage 12–14 Ma [52–54]—carries a beta-type TM, with a two-cysteine motif indicative of non-covalent Env subunit association. In the primate lineage, HERV-K(HML5) is estimated to have infected our ancestors some 55 Ma [55], and also carries a beta-type TM, suggesting a long association with mammals of retroviruses with non-covalently associated Env subunits [26]. Similarly, many examples of gamma-type TM sequences can be found among ERVs estimated to have been infectious tens of millions of years ago, such as an ERV of *Bos taurus*, BoERV1, that is estimated to have integrated into the germline of the ancestors of cattle between 58 and 126 Ma [56]; and the chimpanzee ERV, CERV1, estimated to have integrated as long ago as 82 Myr [57].

**Figure 2.**
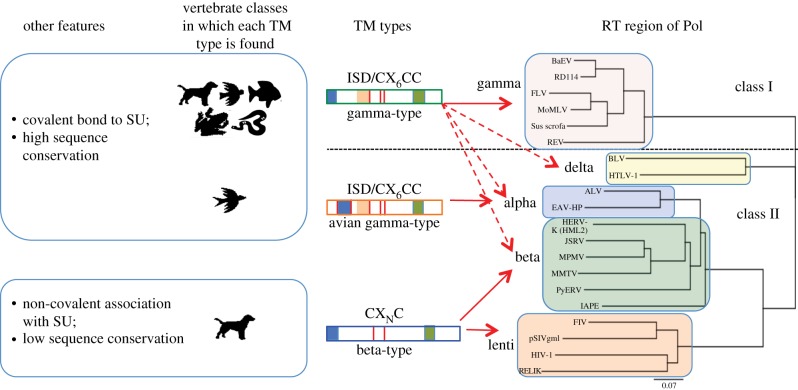
Distribution of TM types among retroviral genera and vertebrate classes. At the right is a neighbour-joining tree based on an alignment of the RT regions of Pol for an assortment of endogenous and exogenous retroviruses. The horizontal dotted line divides class I and class II sequences. The five genera in which these three TM types are found are indicated to the left of the coloured rectangles. In the middle are cartoons representing each TM type; vertical red lines indicate cysteines; blue, orange and green boxes represent the fusion peptide, ISD and the transmembrane region, respectively. Arrows indicate genera in which each TM type is found, with dashed arrows indicating where the gamma-type was acquired via a recombination event. Animal icons represent vertebrate classes—mammals, birds, fishes, amphibians and reptiles.

One of the first indications that *env* has its own story to tell is apparent from the fact that the TM types of several genera do not segregate with the RT classes [16] (figure 2). While retroviruses that cluster with class I ERVs are associated only with the gamma-type TM, retroviruses clustering with class II ERVs are associated with any of the three TM types: lentiviruses, including the known endogenous forms, carry the beta-type TM; deltaretroviruses, the gamma-type TM; and alpharetroviruses, the avian gamma-type variant. The betaretroviral genus is split between the type-B members, which carry the beta-type TM, and the type-D members, carrying the gamma-type TM. These interesting distinctions are concealed in phylogenetic analyses based on RT alone, and illustrate that *env* has its own history independent of *pol*. What dynamic lies behind this distribution of *env* types?

## 4. Env-swapping I

The pattern described above can be explained by recombination events involving acquisition by a retrovirus of a heterologous *env*. Such events are thought to arise from co-encapsidation of two heterologous RNA sequences within the same virion [58]. Interestingly, gamma-type *env* and beta-type *env* differ in their involvement in such recombination events. Incongruencies between phylogenetic trees based on RT and TM sequences of a wide range of ERVs and exogenous retroviruses alike reveal multiple instances of gamma-type *env* acquisition by other gammaretroviruses, as well as by betaretroviruses and other class II retroviruses (excluding the lentiviruses) [16]. By contrast, as yet there are no documented cases of a class I retrovirus, or even another betaretrovirus, acquiring a heterologous beta-type *env*, despite the large number of endogenous betaretroviruses in the genomes of mammals [4,6].

**Figure 3.**
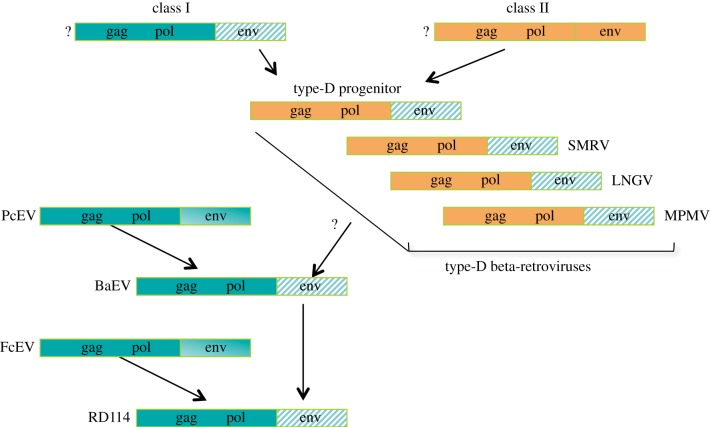
Recombination series involving a gamma-type *env*. Class I viruses are depicted in green; class II viruses, in orange; the ‘promiscuous’ gamma-type *env* is marked with a green diagonal pattern. See text for explanation of question marks and arrows.

A well-characterized case of heterologous *env* acquisition involves a particularly promiscuous gamma-type *env* and the handful of members of the betaretrovirus genus formerly known as the type-D retroviruses (figure 3). Included in this group are MPMV and the closely related simian retroviruses 1 and 2 (SRV-1, -2), squirrel monkey retrovirus (SMRV) and Langur virus (LNGV) [46,59,60]. These viruses appear to be descendants of a virus that infected the primate lineage before the divergence of Old World and New World primates, roughly 50 Ma [61]. The viral progenitor was the product of a recombination event involving class I and class II viruses, by which a betaretrovirus (class II) acquired a gammaretroviral *env* (class I) from an unknown source, then diverged, giving rise to the lineage that includes LNGV and MPMV along the Old World lineage, and SMRV along the New World lineage [61]. Later, a type-D *env* (thus, a gamma-type *env*, even though its donor would be classified as a betaretrovirus) was acquired by a gammaretrovirus known as Papio cynocephalus ERV (PcEV), producing baboon endogenous retrovirus (BaEV) [60]. Thus, a gamma-type *env* passed from a class I to a class II retrovirus, then back to class I again. The chain also extends further in the other direction: the *env* of BaEV recombined with *gag-pol* of Felis catus endogenous virus (FcEV) to produce RD114—an infectious ERV of cats [62].

Another recently discovered example of a class II retrovirus acquiring a gamma-type *env* involves intracisternal type-A particles (IAPs). IAPs are endogenous sequences related to betaretroviruses, and are prevalent in the genomes of various mammals, especially the rodent lineage [63]. While many IAP loci have degraded *env* sequences, others—known as IAPE—have the beta-type *env* typical of class II retroviruses [64]. In a recent study comparing the success of *env*-less IAP lineages with that of IAPE lineages in colonizing genomes, the authors noted that in two independent events, an IAP had acquired a gamma-type *env*, leading to colonization events in the genomes of the guinea pig and the shrew, respectively [65].

Sequence comparisons [66] and incongruency between RT and TM phylogenies [16] indicate that a recombination event, involving acquisition of a gamma-type *env*, gave rise to the Deltaretrovirus genus. The deltaretroviruses (BLV, HTLV-1, -2, -3 and STLV-1) have *gag-pol* genes that cluster among class II retroviruses and share class II features such as the YMDD catalytic domain of RT. The *env* genes, however, cluster with gammaretroviral *env*, and TM in particular has all of the features of the gamma-type. Similarly, members of the Alpharetrovirus genus, consisting of avian leukosis virus (ALV) and its ‘onco-twin’, Rous sarcoma virus, as well as a few closely related viruses of chickens, display incongruence between RT and TM on trees—RT is typical of class II retrovirus, whereas *env* is the avian gamma-type, suggesting a recombinant origin of the Alpharetrovirus genus [16].

In the above cases, we see several instances of the same, or highly similar, gamma-type *env* associated with retroviruses of both class I (PcEV, FcEV and BaEV) and class II (MPMV, SMRV and IAPE), suggesting recombination events. While the examples given above are limited to mammalian species, examples suggesting recombination events involving cross-species transmissions across large genetic distances have also been found. Before describing these, however, it will be helpful to examine the species distributions of *env*, based on the distribution of the corresponding TM types.

## 5. Host range as revealed by endogenous retroviral transmembrane types

Given the divergence between the gamma- and beta-type TM, along with the key role that Env plays in determining host range, we should not be surprised to find a difference in the species distributions of retroviruses with each TM type. Indeed, such is the case, as revealed by an extensive search of the NCBI databases with an array of TM sequences of each type [26]. While the avian gamma-type is specific to birds, the gamma-type TM was found in ERV sequences from at least five classes of vertebrates—mammals, reptiles, amphibians, fish and birds (figure 2*a*). The beta-type, by contrast, was not found in any species in the databases outside the mammalian class. It is noteworthy, however, that within the mammalian class, the beta-type TM was found in a variety of species representing a range of lifestyles, habitats and reproductive features.

While it is true that the content of the databases is heavily biased toward mammals, gamma-type TM sequences, but not a single beta-type TM sequence, were found in at least 26 non-mammalian species. Furthermore, within mammals, the beta-type sequence appeared in 50/52 species in which the gamma-type also appeared. Thus, the findings do not appear to be the result of database bias [26]. The fact that the search was homology based, however, allows for the possibility that any beta-type TM sequences outside mammals may be so diverged as to elude the query sequences. Although this is possible, we think it unlikely given the range of beta-type query sequences used, and the ability of the BLAST algorithm [51] to detect distantly related sequences.

## 6. Env-swapping II

Based on the distribution of TM types among vertebrate species [9,16,26], the host range of the gamma-type TM includes that of beta-type TM (mammals), plus species from four additional vertebrate classes [26]. An implication of this imbalance in host range is that the acquisition of a gamma-type *env* in place of a beta-type *env* could facilitate a cross-species jump between vertebrate classes. Such an event may have occurred in the case of python molurus ERV (PyERV), which has been found in the genomes of two species of pythons [67]. The *gag-pol* region of PyERV clusters with class II ERVs and aligns most closely with betaretroviruses, whereas the *env* is typical of a murine gammaretrovirus. Thus, the acquisition of a gamma-type *env* may have afforded a betaretrovirus access to the reptilian class, regardless of the selective pressure that initially precipitated the recombination.

Interestingly, another recombinant ERV comprising a class II *gag-pol* region with a gamma-type *env* was recently discovered in the genomes of several avian species [3,68]. In this case, the *gag-pol* region has features of both alpha- and betaretroviruses, and in phylogenetic analyses is positioned basal to the alpharetroviral clade. This situation raises the intriguing possibility that the alpharetroviral lineage arose from a class II progenitor that, owing to its acquisition of a gamma-type *env*, was able to infect several avian species. At some point subsequent to the initial infection, the gamma-type *env* would have been replaced by the avian gamma-type variant.

The avian gamma-type *env*, such as the gamma-type *env*, has itself been the target of capture; the ALV-J subgroup of ALV has an *env* that differs significantly from those of the remaining subgroups (A, B, D and E) and was acquired by recombination with an endogenous provirus [69]. As more avian genomic sequences are added to the databases, more such cases will likely emerge.

Remarkably, the avian gamma-type TM is strikingly similar in structure and features to fusion glycoproteins found in two other virus families, the filoviruses and the arenaviruses [16,50,70]. The ALV TM sequence (excluding the CT) is 27% identical and 42% similar at the amino acid level to both Marburg virus and Ebola virus. Furthermore, the two filoviruses share with alpharetroviruses a conserved CX_6_CC motif, a recognizable ISD region, a fusion peptide that is flanked by a pair of cysteines, and a highly conserved predicted N-glycan site at the start of the first heptad repeat (hr1). While it is possible that filoviruses originated in mammals, it is noteworthy that the cysteine-flanked fusion peptide specific to the avian gamma-type TM has not yet been seen in any retroviral TM outside the avian class, raising the intriguing possibility that Ebola virus and Marburg virus originated in an avian species.

In the case of the arenaviruses, an intriguing parallel to PyERV (whereby acquisition of a gamma-type *env* affords a mammalian class II retrovirus access to a new host class) is presented. Arenaviruses were previously thought to be limited to mammals. However, two arenavirus strains were recently isolated from tree boas and boa constrictors suffering from inclusion body disease [70]. The authors of the study were surprised to find that the glycoprotein sequences diverged greatly from those of the known mammalian arenaviruses, and more closely resembled those of Ebola virus and ALV. In fact, an alignment shows that the ALV TM sequence is 27–29% identical and 49–50% similar to the fusion glycoproteins (GP2) of the two arenavirus strains. Again, these sequences share with ALV a CX_6_CC motif, the predicted N-glycan positioned at the start of hr1, and an fp sequence that is flanked by cysteines. The ISD sequence seen in gamma- and avian gamma-type TM, however, is not recognizable in the arenavirus sequences.

## 7. Divergence rates differ markedly between the gamma-type and beta-type transmembrane

From the fact that gamma-type TM sequences are found among multiple vertebrate classes, whereas the beta-type is limited to mammals, one might expect the gamma-type to be more divergent as a group than the beta-type. In fact, the opposite is seen: the gamma-type TM sequences are marked by a high average pairwise identity at the amino acid level, demonstrating low overall divergence, whereas the beta-type TM group as a whole is very diverse, as shown by low levels of average pairwise identity (table 1).

**Table 1.** Divergence rates among TM types.

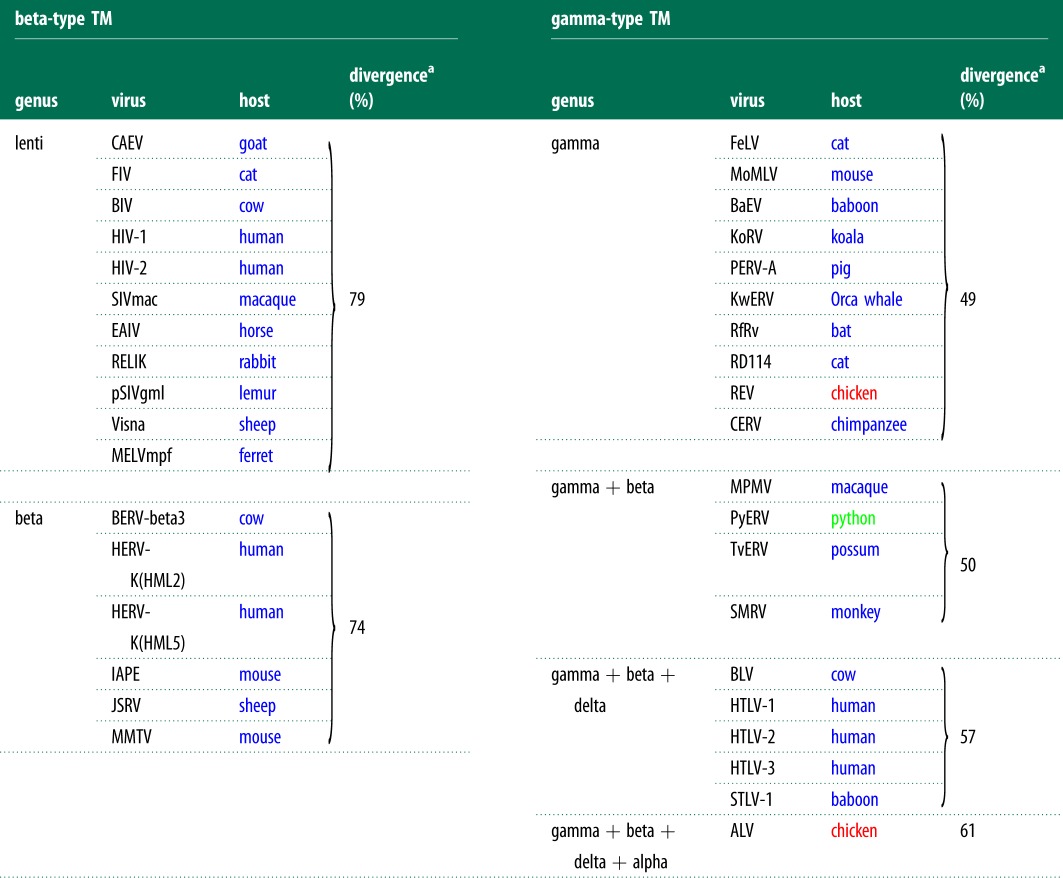

^a^As measured by (1 − average pairwise identity) at the amino acid level; blue font represents the mammalian class; red, the avian class; green, the reptilian class.

A collection of lentiviruses, for example, including the known endogenous forms, represents just one vertebrate class and one retroviral genus, yet has an average pairwise identity of just 21% among TM sequences. Similarly, among beta-type TM sequences, we see that a collection of six endogenous and exogenous TM sequences that represent only one class (mammals) and one genus (betaretroviruses) has an average pairwise identity of 26%. In stark contrast, within the gammaretroviral genus, a group of 10 TM sequences representing both exogenous and endogenous members from two vertebrate classes has an average pairwise identity of 51%—nearly twice that of the betaretrovirus genus. Adding TM sequences from four type-D betaretroviruses gives an average of 50%, in keeping with their having acquired a gammaretroviral *env*. Note that this collection represents three vertebrate classes and two retroviral genera, yet exhibits far less diversity than either the betaretroviral genus or the lentiviral genus.

In fact, even including TM sequences from two additional genera—the delta- and the alpharetrovirus genera, thus spanning four retroviral genera and three vertebrate classes—the average pairwise identity of this group (39%) still well exceeds that of either of the genera possessing the beta-type TM.

Although the high degree of variability in the SU region of *env* makes it difficult to carry out a comparison of divergence similar to that carried out for TM, a couple of observations are worthy of comment. Among alpharetroviruses and the murine gammaretroviruses, a pattern is seen in SU amino acid sequences wherein a few discrete variable regions are flanked by regions of relatively high conservation [71,72]. In many cases, such as with ALV-A and -B variants, the two variants have only a few amino acid changes in the variable region, yet bind cognate receptors that are quite different from one another in sequence and structure [73]. This situation suggests a strategy in which mutational space can be explored via a small variable region within the context of a well-conserved structure. Similarly, within the variants of MLV (i.e. ecotropic, amphotropic, xenotropic), SU has discrete variable regions near the N-terminus, where the RBD is found, with a rather highly conserved (64% identity at the amino acid level) C-terminal portion consisting of a proline-rich region (PRR) and the C-terminal domain (CTD) [74]. Contrast this situation with that of the primate lentiviruses, where SU varies greatly among different isolates despite their use of the same receptor (CD4) [73].

Thus, the relative rates of divergence of SU appear to mirror those of TM for the gamma-type and beta-type *env*. This contrast in overall divergence within each type further supports the idea of independent origins or at least divergence from a common progenitor far back in evolutionary time.

## 8. Concluding remarks

The story revealed by TM sequences in the ERV fossil record is one of significant differences hidden beneath a veneer of similarity. The TM sequences of all *Orthoretrovirinae*—excluding the epsilonretroviruses—share a specific domain organization and certain features such as a cysteine pair in the ectodomain, heptad repeats, a fusion peptide and a transmembrane region. Given this level of conservation, it is all the more striking to discover such intriguingly different evolutionary ‘lifestyles’ of retroviral envelope glycoproteins.

On the one hand is the tightly honed gamma-type, maintaining a restrained form while nevertheless adapting to species from five vertebrate classes. Furthermore, the gamma-type and its avian variant have participated in multiple recombination events, leading to expansion beyond the class I gammaretroviruses to class II retroviruses—to deltaretroviruses, alpharetroviruses and some betaretroviruses. In some cases, acquisition of a gamma-type *env* appears to have allowed a class II retrovirus to ‘hitchhike’ into another vertebrate class. Even more remarkably, we see envelope glycoproteins homologous to the avian gamma-type in two additional virus families—the filoviruses and the arenaviruses.

In sharp contrast to the gamma-type is the beta-type, which presents a much more divergent set of sequences, loosely casting about in mutational space to adapt to a relatively narrow range of species and receptors. In further contrast to the promiscuous gamma-type *env*, the beta-type *env* has yet to be implicated in an *env* acquisition event. Beta-type *env* has only been found among class II ERVs and their exogenous relatives (specifically, the lentiviruses and the non-recombinant betaretroviruses) and only within the mammalian class.

The distinct life histories presented by the beta- and gamma-types lead to some fresh lines of inquiry. How does the divergence profile of the gamma-type relate to its success across multiple vertebrate classes? And what prevents the beta-type from branching out of the mammalian class, despite enjoying wide-ranging success within its limits? Do the differing mechanisms of subunit association impact each type's ability to adapt? In the gamma-type *env*, the subunits are joined via a single covalent bond involving a specific motif in TM—the CX_6_CC region—to a cysteine in the CTD of SU. One could speculate that this configuration is key in allowing the remainder of SU to evolve a highly effective modular organization, whereby a variable RBD is positioned at the N-terminus, followed by a PRR that may serve as a flexible arm [73]. Under this model, the structure would afford the RBD a freedom of movement that increases its efficiency in exploring interactions with novel receptors. By contrast, in the beta-type *env* of HIV-1, weak interactions between SU and TM occur over multiple residues at both the N-terminal and C-terminal regions of SU [23]—a configuration that may limit the flexibility with which the beta-type *env* can explore mutational space.

Why have we seen multiple cases of class II viruses acquiring a gamma-type *env*, but not the converse? Are there structural features or other obstacles to fitness that prevent gammaretroviruses from acquiring beta-type *env*? Regardless of the reasons behind the ability of the gamma-type *env* to infect a wide range of species from multiple vertebrate classes, it is likely that ‘success breeds success’. In other words, the wider host range offered by a gamma-type *env* would be expected to drive recombination events such that the gamma-type is favoured over the beta-type—a dynamic that is supported by the ERV fossil record, with the beta-type seeming to lose ground to the gamma-type. And given the evidence for several betaretroviruses having acquired a gamma-type *env*, is it possible that a lentivirus could do the same? What would be the implications for host range and pathogenicity of such a recombinant?

The advantages afforded by acquisition of a gamma-type *env* need not be limited to the ability to bind novel receptors, but could extend to other functions of Env, such as the immunosuppression mediated by the ISD, which could confer more robust infectivity, or changes in infection kinetics or pathogenicity that favour endogenization. Although the ISD of some murine and primate retroviruses has been shown to modulate immunity *in vivo* [34–36], it is unknown to what extent it retains this function in other vertebrate classes, suggesting another interesting line of inquiry.

In conclusion, one chapter of the story that *env* tells through the fossil record goes something like this: when a betaretrovirus acquires a gamma-type *env*, regardless of the selective pressure that drives the recombination event, it acquires the chance of accessing a new niche, with new selective pressures on the entire virus. In the process of adapting to the new host, the virus will diverge from its recombinant progenitor, probably all the more so in cases involving cross-species jumps between vertebrate classes. Although such events appear to be rare [75], they can have significant impact—generating new viral lineages, and even new genera. In this context, the differences between gamma-type and beta-type *env* described here suggest that the broad division between class I and class II ERVs based on RT sequences represents divergence that was driven by association with two very different *env* types—class I with the gamma-type *env*, and class II with the beta-type *env*. These two *env* types developed different dynamics as they followed different paths, with the gamma-type acquiring modularity that may have contributed to its wide host range, and the beta-type limited to mammals. The propensity for recombination shown by the gamma-type, in conjunction with its wider host range, may have begun driving recombination towards replacement of the beta-type *env* with gamma-type *env*, with these events seeding new genera among class II viruses—the deltaretroviruses, the alpharetroviruses and the type-D betaretroviruses.

## 9. Material and methods

### (a) Phylogenetic analysis

Alignments and trees were generated in Geneious v. 6.0.4, created by Biomatters, available from http://www.geneious.com, using the ClustalW algorithm [76]. The neighbour-joining tree depicted in figure 3 was generated from an alignment of 177 amino acids spanning the RT domain of *pol*. Divergence values in table 1 were calculated based on average pairwise identities from alignments of TM sequences beginning at the SU–TM cleavage site and extending through the transmembrane region.

### (b) Viruses and accession numbers

ALV, avian leukaemia virus, NC_015116.1; BaEV, AF142988.1; BERV-beta3, bovine ERV-beta3, EF030818.1; BIV, bovine immunodeficiency virus, L04972.1; BLV, bovine leukemia virus, NC_001414.1; CAEV, caprine arthritis encephalitis virus, NC_001463.1; CERV, chimpanzee endogenous retrovirus, http://saturn.adarc.org/paleo/site/html/CERV-1.html; EAV-HP, NC_005947.1; EIAV, equine infectious anemia virus, M16575.1; FeLV, feline leukemia virus, NC_001940.1; FIV, feline immunodeficiency virus, NC_001482.1; HERV-K (HML2), human endogenous retrovirus-K (HML2) subfamily, JN675087.1; HIV-1, human immunodeficiency virus-1, NC_001802.1; HIV-2, AF082339.1; HTLV-1, -2, -3, human T-lymphotropic virus-1, NC_001436.1; -2, NC_001488.1, -3, DQ093792; IAPE, intracisternal A-type particles elements with an envelope, M73818.1; JSRV, Jaagsiekte sheep retrovirus, NC_001494.1; KoRV, Koala retrovirus, AF151794.2; KwERV, killer whale endogenous retrovirus, GQ222416.1; MPMV Mason–Pfizer monkey virus, NC_001550.1; MoMLV, Moloney murine leukemia virus, NC_001501.1; MMTV, mouse mammary tumor virus, NC_001503.1; MELVmpf, Mustelidae endogenous lentivirus mustela putorius furo, http://saturn.adarc.org/paleo/site/html/MELVmpf.html; PERV-A, porcine endogenous retrovirus-A, EU789636.1; pSIVgml, primate SIV grey mouse lemur, FJ461357.1 (Pol), FJ461356.1 (Env); PyERV, python molurus ERV, AAN77283.1 (Pol), AAN77282.1 (Env); RELIK, rabbit endogenous lentivirus-K, FJ493031.1 (*pol*), FJ493038.1 (*env*); RD114, AB674443.1; REV, reticuloendotheliosis virus, NC_006934.1; RfRV, Rhinolophus ferrumequinum retrovirus, JQ303225.1; SIVmac, simian immunodeficiency virus, AAC12636.1; STLV-1, simian T-lymphotropic virus-1, NC_000858.1; SMRV, squirrel monkey retrovirus, M23385.1; TvERV, trichosurus vulpecula ERV, AF284693.1; Visna maedi virus, AAA48362.1 (Env); AGTQ01068359.1 (Pol).
